# An Enhanced Paradigm for Cognitive Cooperation Networks: Two-to-One Energy and Spectrum Dual-Cooperation

**DOI:** 10.3390/s18072085

**Published:** 2018-06-29

**Authors:** Zhihui Liu, Wenjun Xu, Junyi Wang, Tao Dong, Hongbing Qiu

**Affiliations:** 1 State Key Laboratory of Space-Ground Integrated Information Technology, Beijing Institute of Satellite Information Engineering, Beijing 100095, China; liuzhihui1225@163.com (Z.L.); dongtaoandy@163.com (T.D.); 2 Key Lab of Universal Wireless Communications, Ministry of Education, Beijing University of Posts and Telecommunications, Beijing 100876, China; 3 Guangxi Key Laboratory of Wireless Wideband Communication and Signal Processing, Guilin University of Electronic Technology, Guilin 541004, China; wangjy@guet.edu.cn (J.W.); qiuhb@guet.edu.cn (H.Q.)

**Keywords:** energy and spectrum dual-cooperation, cognitive cooperation networks, spatial multiplexing, network coding

## Abstract

In this paper, two-to-one energy and spectrum dual-cooperation (ESDC) is investigated for cognitive cooperation networks. Specifically, the energy and spectrum of two primary users (PUs) are both transferred or authorized to one multi-antenna secondary user (SU) in exchange for its aid in the signal relaying to guarantee the successful data transmission, whilst the SU, which originally owns no spectrum access privilege and limited energy storage, is also able to concurrently transmit its own data through spatial multiplexing. Moreover, network-coding is also adopted to further compress the data size and hence reduce the power consumption at SU. The formulated problem for the aforementioned two-to-one ESDC model is non-convex and intractable to solve directly. To solve the problem effectively, the Lagrangian dual methods plus fixed-point iteration methods and semidefinite relation methods are employed, and the optimal solution could be achieved through iterative optimization. Simulation results show that, compared with the traditional spectrum-only cooperation, the proposed two-to-one ESDC paradigms can greatly improve the successful transmission probability for PUs and achievable transmission rate for SU. Meanwhile, the proposed two-to-one dual-cooperation modes are significantly superior to the one-to-one cooperation mode, in terms of spectrum efficiency and energy efficiency.

## 1. Introduction

Along with the rapid development of mobile communication systems and the springing up of new wireless applications, the energy consumption issues of wireless networks have increasingly attracted attention from academia and industry [1,2,3]. Owing to shortening the actual communication distances (i.e., multi-hop cooperation) [4] or boosting the potential wireless channels (i.e., multi-point cooperation) [5], cooperative communication has the capability of decreasing the overall system energy cost while guaranteeing the prescribed transmission targets, and can be deemed as a kind of green communication paradigm [6].

The explosive growth of wireless traffic volume also causes the severe shortage of spectrum resources, and hence cognitive radio (CR) has been proposed to efficiently solve the problem of spectrum scarcity [7]. As one of the most successful application modes for CR, cognitive cooperation, which is capable of combining the superiorities of both CR and cooperation, is becoming a research hotspot [8,9,10]. The basic idea of cognitive cooperation is spectrum cooperation also referred to asspectrum lease, between primary users (PUs) and secondary users (SUs), resulting in a win–win solution for both sides. That is, SUs are allowed to utilize the licensed spectrum resource, in exchange for assisting PUs in forwarding data to the intended receivers. In return, SUs can also transmit their own data over the licensed spectrum, in the premise that the transmission requirements for PUs are strictly satisfied.

Via spectrum cooperation, a spectrum resource is authorized to SUs for cooperative transmission opportunities. By revisiting the Shannon’s capacity formula C=Wlog(1+P/N) [11], it is observed that, in order to strengthen the cooperative transmission ability of SUs, aside from the spectrum cooperation to increase the bandwidth *W*, raising the signal power *P* may be another approach to attain the higher capacity *C*. This inspires the basic concept of energy cooperation, i.e., a portion of PUs’ energy is transferred to SUs for cooperative transmission opportunities. In general, the energy transfer process can be wireless or wired [12,13]. For convenience, however, wireless power transfer (WPT) is preferred to support remote energy charging for SUs in a more steerable manner [14]. Through energy cooperation, energy resources are appropriately transferred from PUs to SUs, and the latter usually owns the better transmission conditions for the receivers, i.e., primary receivers (PRs) and secondary receivers (SRs). Thus, energy cooperation enables the energy trade-off between primary transmission and secondary transmission, and has the potential to achieve the higher energy efficiency.

SUs are often more energy-hungry in cognitive cooperation networks, due to not only data transmission for themselves, but also data forwarding for PUs. In addition, energy cooperation and spectrum cooperation can increase the transmission capacity of SUs from two distinct points of supplementing the bandwidth *W* and the signal power *P*, respectively. Energy and spectrum dual-cooperation (ESDC) is beginning to be studied for CR networks [15,16]. The authors in [15] first study this kind of dual-cooperation, and simultaneous wireless information and power transfer (SWIPT) is adopted to harvest energy from radio frequency (RF) signals along with traditional information reception [17]. In [15], the energy harvesting at the SU is implemented using either time splitting (TS) or power splitting (PS) [18,19]. They derive the optimal beamforming solution at the SU, and further verify that the PS protocol can achieve a larger rate region than the TS protocol. The work [16] further proposes a novel system model based on the TS-plus-PS protocol, and maximizes the achievable throughput of the SU by jointly optimizing time-division proportions, the PS factor and the transmit vectors at the SU.

The above research is confined to the one-to-one cooperation model, where one PU and one SU come into the agreement about how to perform spectrum allocation and energy transfer to satisfy the individual benefits, i.e., the rate requirements. In practical CR scenarios, the SU is usually able to cooperate with multiple PUs simultaneously to obtain the more energy and spectrum resource replenishment, especially when the SU is equipped with multiple antennas and enabled to receive/transmit signals from/to multiple PUs through spatial multiplexing [20,21]. Therefore, in this paper, an enhanced three-party (two PUs and one multi-antenna SU) cooperation model is considered, referred to as two-to-one ESDC model. Compared to the existing one-to-one model, such a two-to-one cooperation model will explicitly lead to the greener networking, since the energy and spectrum balance among three parties, no longer two parties, is empowered to increase the energy efficiency considerably. We will study how the multi-antenna SU performs ESDC effectively by using spectrum leasing and power splitting, i.e., how to design the PS factor and beamforming vectors to make the best use of the cooperated energy and spectrum resource.

First of all, different from our previous work of amplify-and-forward cooperative mode [22], we focus on the decode-and-forward (DF) cooperative mode [23], i.e., the information of two PUs is first decoded, and then forwarded, with SU’s energy and harvested energy, to the intended receivers through different precoding vectors. The considered problem is formulated with the aim of maximizing the achievable transmission rate of the SU by optimizing the PS factor and precoding vectors at the SU, provided that the minimum transmission rate requirements are guaranteed for both PUs and the total power consumption at the SU is not more than the available power, i.e., the maximal battery power plus the harvested power from RF signals. By analyzing the characteristic of the problem at hand, and leveraging dual methods as well as fixed-point iteration methods, the optimal solutions are derived for this two-to-one ESDC problem.

Moreover, inspired by the idea of network coding (NC) [24,25,26], in the case of insufficient spatial degree of freedom (DoF) and symmetrical primary transmissions, an NC-assisted two-to-one ESDC model is further proposed for cognitive cooperation networks to improve the cooperative transmission capability, and hence increase the energy utilization efficiency. Specifically, for symmetrical primary transmissions, two primary transmit–receive pairs form a butterfly network topology, and NC is adopted at the secondary transmitter, i.e., recoding the decoded data of two PUs into one stream through the exclusive or (XOR) operation. operation and broadcasts it to two primary receivers, to further reduce the energy consumption for primary signals’ forwarding. For this formulated problem, the semi-definite programming (SDP) and rank-reduction technology are employed to combat the intractable non-convexity and achieve the optimal PS factor and precoding vectors.

Note that the two-to-one spectrum-only cooperation model has been preliminarily studied in our previous work [20,21] for flat-fading channel model and spectrum-selective channel model respectively, in which the frequency-division cooperation [27] is used to improve the transmission quality of service (QoS) for both PUs and SU. The authors in [28,29] have also investigated the spectrum leasing problem in a similar spectrum-only cooperation model. The work [28] focuses on designing the cooperative strategy at the side of PUs based on the frequency-division cooperation, while [29] concentrates on how to select cooperative SUs for each PU and how to design time allocation for each authorized spectrum band on a basis of the time-division cooperation [30]. In this paper, an aggressive cooperation model, i.e., two-to-one simultaneous energy and spectrum cooperation, is taken into account to boost the system spectrum efficiency and energy efficiency. Moreover, the space–division cooperation [31] is adopted to fully exploit the shared energy and spectrum resource, where the SU, equipped with multiple antennas, simultaneously relays PUs’ data and transmits its own data on the same frequency band through spatial multiplexing. Furthermore, different from amplify-and-forward (AF)-based two-to-one simultaneously energy and spectrum cooperation, an enhanced two-to-one simultaneously energy and spectrum cooperation based on AF and NC considering topology characteristics is investigated.

The main contributions of this paper are summarized as follows.

1. **The two-to-one ESDC model is established based on spatial multiplexing, and the optimal cooperation approach is proposed**. In our model, the SU simultaneously cooperates with two PUs to obtain more energy and spectrum resource from PUs, which consequently improves the transmission quality of both PUs and SU. For this considered model, both the energy cooperation strategy, i.e., selecting a proper PS factor, and the spectrum cooperation strategy, i.e., deriving suitable precoding vectors for both PUs’ data relaying and SU’s data delivery, should be jointly optimized. However, by analyzing the impact of the PS factor on data reception/forwarding in two slots, the energy cooperation and spectrum cooperation are effectively decoupled without loss of optimality, and eventually the optimal solution to the energy cooperation and spectrum cooperation is achieved by using dual methods as well as fixed-point iteration methods.
2. **The NC-assisted two-to-one ESDC model is established for symmetrical primary transmissions, and the optimal cooperation approach is also proposed**. For symmetrical primary transmissions, network coding is further introduced to compress two PUs’ data stream into one and hence reduce the energy consumption. Likewise, the considered problem is optimally decoupled to degrade the intractability of dual-cooperation optimization. Furthermore, by resorting to the SDP and rank-reduction methods to overcome the non-convexity of the decoupled problem, the optimal energy and spectrum cooperation approach is achieved for this extended model as well.

The rest of this paper is organized as follows. Section 2 describes the system model and problem formulation for the two-to-one ESDC in cognitive cooperation networks, and Section 3 presents the optimal algorithm design in detail. Then, in Section 4, we further consider the NC-assisted two-to-one ESDC model and propose the optimal cooperation approach. Simulation results are provided in Section 5, and finally the paper is concluded in Section 6.

**Notations**. Throughout this paper, vectors and matrices are represented by boldface lowercase and uppercase letters, respectively. ∥·∥, ${\left(\cdot \right)}^{T}$, ${\left(\cdot \right)}^{-1}$, ${\left(\cdot \right)}^{H}$ and ⊙ represent the Frobenius norm, transpose, inverse, Hermitian transpose and element-wise product operations of vectors or matrices, respectively. A⪰0 means that A is positive semi-definite. I and 0 denote an identity matrix and an all-zero matrix, respectively, with appropriate dimensions. $C^{x\times y}$ denotes the space of x×y complex matrices. R denotes the set of real numbers. E(·) denotes the statistical expectation of the argument. Tr(·) and Rank[·] denote the trace operator and the rank of the argument, respectively. The notation x∼CN(v,Σ) means that x is a random vector following a complex circular Gaussian distribution with mean v and covariance Σ.

## 2. System Model and Problem Formulation for Generalized Energy and Spectrum Dual-Cooperation

In this section, a generalized two-to-one cognitive cooperation system model is investigated with energy and spectrum dual-cooperation, followed by the corresponding problem formulated.

### 2.1. A Generalized System Model for Two-to-One Cognitive Cooperation

**Figure 1:**
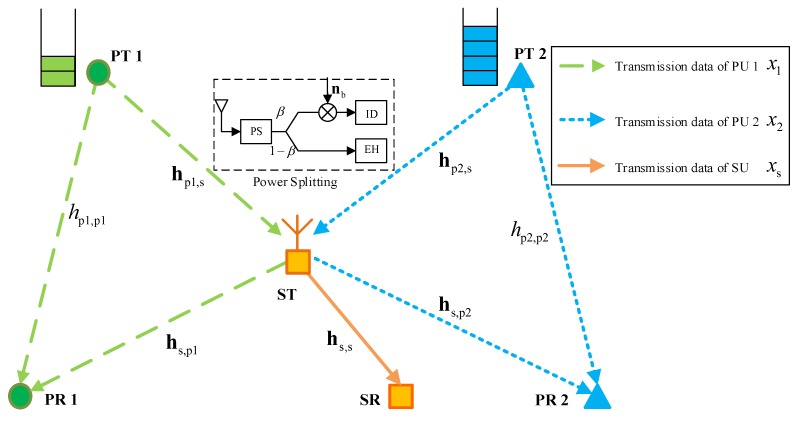
System model of the two-to-one energy and spectrum dual-cooperation in cognitive cooperation networks.

As shown in Figure 1, the considered two-to-one cognitive cooperation system consists of one secondary transmit-receive pair and two primary transmit-receive pairs, i.e., (ST, SR) and (PT *i*, PR *i*), i=1,2, with ST denoting secondary transmitter and PT denoting primary transmitter. PTs, PRs and SR are all equipped with one antenna, while ST is equipped with *N* antennas. For brevity, the transmission channels involved in the studied model are defined as follows. $h_{pi,s}$, $h_{pi,pj}$ are transmission channel gains from PT *i* to ST and PR *j* respectively, and $h_{s,s}$, $h_{s,pj}$ are transmission channel gains from ST to SR and PR *j*, respectively, where i,j=1,2.

In our research, it is assumed a joint spectrum and energy cooperation agreement is established between PUs and SUs. In the established agreement, ST receives RF signals from PTs in the first slot to execute the signal decoding and energy harvesting simultaneously by using the PS protocol, and jointly transmits both PUs’ data and its own data in the second slot through spatial multiplexing.

The whole transmission is completed in two slots. In the first slot, two PTs broadcast their message to both PRs and ST. The received signal at PRs and ST, respectively, are

(1) $$\begin{array}{ccc} y_{\mathrm{PR}j,1} & = & \sum_{i=1,2}h_{pi,pj}\sqrt{P_{i}}x_{i}+n_{pj},\;j=1,2, \end{array}$$

(2) $$\begin{array}{ccc} y_{\mathrm{ST}} & = & \sum_{i=1,2}h_{pi,s}\sqrt{P_{i}}x_{i}+n_{a}, \end{array}$$

where $x_{i}$ and $P_{i}$ are the normalized transmission data and transmit power at PT *i*, and $n_{pi}∼CN\left(0,\sigma_{p}^{2}\right)$ is the received noise at PR *i*, where i=1,2. $n_{a}∼CN\left(0,\sigma_{a}^{2}I\right)$ is the received noise at ST from antennas.

After receiving $y_{\mathrm{ST}}$, ST inputs it into the power splitter to diverge into two legs. One is used for energy harvesting (EH), and the harvested energy is

(3) $$\begin{array}{c} Q\left(\beta \right)=\eta \left(1-\beta \right)(\sum_{i=1,2}P_{i}∥h_{pi,s}{∥}^{2}+N,\sigma_{a}^{2}), \end{array}$$

where β∈(0,1] is the PS factor and η∈(0,1] is energy conversion efficiency. The other part is for information decoding (ID), and the final observation at ID can be expressed as

(4) $$\begin{array}{c} r_{\mathrm{ST}}=\sqrt{\beta }y_{\mathrm{ST}}+n_{b}=\sqrt{\beta }\sum_{i=1,2}h_{pi,s}\sqrt{P_{i}}x_{i}+\sqrt{\beta }n_{a}+n_{b}, \end{array}$$

where $n_{b}∼CN\left(0,\sigma_{b}^{2}I\right)$ is the additive noise incurred during the RF/Baseband conversion and the following baseband signal processing.

At ID, the minimum mean square error (MMSE) method is adopted to realize linear signal detection, and the receiving rate of PU *i*’s transmission data in the first slot can be written as

(5) $$\begin{array}{c} R_{pi,1}=\frac{1}{2}\Delta f\log_{2}\left(1+\gamma_{pi,1}\right),\;\gamma_{pi,1}=P_{i}h_{pi,s}^{H}A_{i}^{-1}h_{pi,s}, \end{array}$$

where $\gamma_{pi,1},i=1,2$ is the received signal to interference plus noise ratio (SINR) for PU *i*’s signal at ST, $A_{i}=P_{j}h_{pj,s}h_{pj,s}^{H}+\left(\sigma_{a}^{2}+\sigma_{b}^{2}/\beta \right)I,j\neq i$.

In the second slot, ST transmits data to PR 1, PR 2, and SR simultaneously through spatial multiplexing, and the precoding vectors for PU 1, PU 2, and SU are $w_{1}\in C^{N\times 1}$, $w_{2}\in C^{N\times 1}$, and $w_{3}\in C^{N\times 1}$, respectively. Thus, the transmitted signal at ST is $s=w_{1}x_{1}+w_{2}x_{2}+w_{3}x_{s}$ with $x_{s}$ denoting the normalized transmission data of SU. As a result, the actually consumed power at ST can be expressed as

(6) $$\begin{array}{c} P_{c}=∥w_{1}{∥}^{2}+∥w_{2}{∥}^{2}+{∥w_{3}∥}^{2}-Q\left(\beta \right). \end{array}$$

Meanwhile, PR *i* receives the transmitted signal s, and endeavors to decode its intended signal $x_{i}$. The received signal at PR *i* is

(7) $$\begin{array}{c} y_{\mathrm{PR}i,2}=h_{s,pi}^{H}s+n_{pi}=h_{s,pi}^{H}\left(w_{1}x_{1}+w_{2}x_{2}+w_{3}x_{s}\right)+n_{pi} \end{array}$$

and combined with the received signal in the first slot, i.e., Equation (1), the ultimate receiving rate at PR *i* after maximum-ratio-combination (MRC) can be calculated as

(8) $$\begin{array}{ccc} R_{pi,2} & = & \frac{1}{2}\Delta f\log_{2}\left(1+\frac{P_{i}{\left|h_{pi,pi}\right|}^{2}}{P_{j}{\left|h_{pj,pi}\right|}^{2}+\sigma_{p}^{2}}+\gamma_{Pi,2}\right), \end{array}$$

(9) $$\begin{array}{ccc} \gamma_{pi,2} & = & \frac{|h_{s,pi}^{H}w_{i}{|}^{2}}{|h_{s,pi}^{H}w_{j}{|}^{2}+{\left|h_{s,pi}^{H}w_{3}\right|}^{2}+\sigma_{p}^{2}}, \end{array}$$

where j≠i,i=1,2.

Similarly, SR receives s and decodes $x_{s}$ from it. The received signal and receiving rate can be calculated as

(10) $$\begin{array}{ccc} y_{\mathrm{SR}} & = & h_{s,s}^{H}s+n_{s}=h_{s,s}^{H}\left(w_{1}x_{1}+w_{2}x_{2}+w_{3}x_{s}\right)+n_{s}, \end{array}$$

(11) $$\begin{array}{ccc} R_{s} & = & \frac{1}{2}\Delta f\log_{2}\left(1+\gamma_{s}\right),\gamma_{s}=\frac{|h_{s,s}^{H}w_{3}{|}^{2}}{\sum_{i=1,2}{\left|h_{s,s}^{H}w_{i}\right|}^{2}+\sigma_{s}^{2}}. \end{array}$$

### 2.2. Problem Formulation for Generalized Two-to-One Cognitive Cooperation

In the studied energy and spectrum dual-cooperation model, two PUs and one SU reach the three-party agreement to benefit all participants. In detail, PUs benefit from a guarantee of their minimum required transmission rate, and SU acquires the opportunity to transmit its own data. Mathematically, we formulate the problem as follows:

(12) $$\begin{array}{cc} Q_{0}: & \max_{w_{i},\beta }R_{s}\\ s.t. & C_{1}:R_{pi,1}\geq R_{pi,\mathrm{th}},\;i=1,2,\\ & C_{2}:R_{pi,2}\geq R_{pi,\mathrm{th}},\;i=1,2,\\ & C_{3}:P_{c}\leq P_{\mathrm{th}}, \end{array}$$

where $R_{pi,\mathrm{th}}$ is the minimum transmission data requirement for PU *i*, and $P_{\mathrm{th}}$ is the actually available transmit power at ST.

In this problem formulation, we aim at maximizing the achievable transmission rate for SU by jointly optimizing the PS factor and precoding vectors at ST subject to the minimum rate requirements for two PUs and the actually consumed power constraint at ST. For convenience of expression, define $z_{pi,1}=2^{2R_{pi,\mathrm{th}}/\Delta f}-1$, $z_{pi,2}=z_{pi,1}-\frac{P_{i}{\left|h_{pi,pi}\right|}^{2}}{P_{j}{\left|h_{pj,pi}\right|}^{2}+\sigma_{p}^{2}}$. Problem $Q_{0}$ can be rewritten as

(13) $$\begin{array}{cc} Q_{0}: & \max_{w_{i},\beta }\gamma_{s}\\ s.t. & C_{1}:\gamma_{pi,1}\geq z_{pi,1},\;i=1,2,\\ & C_{2}:\gamma_{pi,2}\geq z_{pi,2},\;i=1,2,\\ & C_{3}:P_{c}\leq P_{\mathrm{th}}. \end{array}$$

**Remark** **1.** Due to the limited transmit power at PTs and ST and the minimum data requirements for PUs, the formulated problem $Q_{0}$ may be infeasible. In the following, the algorithm design is explained only for the feasible case. In the case of infeasibility, the established three-party agreement is feeble and the formulate problem is of no sense. Nevertheless, to verify the superiority of the proposed ESDC scheme, the probability that the formulated problem is feasible, referred to as successful transmission probability for PUs, is also plotted in the simulations, as shown in Figures 3–8.

## 3. Algorithm Design for Generalized Energy and Spectrum Dual-Cooperation

### 3.1. Optimal PS Factor for Energy Cooperation

Through close observation of problem $Q_{0}$, it is found that $\gamma_{pi,1}$ is a monotonic increasing function about β as shown in Equation (5), and Q(β) is a monotonic decreasing function about β as shown in Equation (3). Hence, there is a trade-off of the value of PS factor to balance the signal decoding in the first slot and data delivery in the second slot: on one hand, due to the limitation of transmit data capability from PT *i* to ST in slot one, ST may not be able to successfully decode PT *i*’s transmission data if a large proportion of signal power is used for energy harvesting. On the other hand, the available transmit power at ST is small in the case of a small proportion of signal power used for energy harvesting, and hence PRs may not be able to successfully decode their intended signal due to the limited data forwarding capability of ST.

This trade-off reveals that ST should harvest as much energy as possible to improve its data transmitting capability in the second slot on the premise that the reserved signal energy is sufficient to decode the transmitted data from two PTs. As a result, the optimal PS factor $\beta^{★}$ at ST can be calculated as

(14) $$\begin{array}{ccc} \beta^{★} & = & \max\{\beta_{\min,1},\beta_{\min,2}\},\\ \beta_{\min,i} & = & \min\{\beta \in \left(0,1\right]|\gamma_{pi,1}\geq z_{pi,1}\}. \end{array}$$

$\beta_{\min,i}$ is minimum β with which the received SINR of PU *i*’s data reaches its prescribed threshold $z_{pi,1}$. Although there is no closed-form expression for $\beta_{\min,i},i=1,2$, it is fortunate to find that $\gamma_{pi,1}$ is a monotonically increasing function about β, as shown in Equation (5). As a result, $\beta_{\min,i},i=1,2$ can be effectively found by using the bisection search method.

After obtaining the optimal $\beta^{★}$, problem $Q_{0}$ can be reformulated as

(15) $$\begin{array}{cc} Q_{1}: & \max_{w_{i}}\frac{|h_{s,s}^{H}w_{3}{|}^{2}}{\sum_{i=1,2}{\left|h_{s,s}^{H}w_{i}\right|}^{2}+\sigma_{s}^{2}}\\ s.t. & C_{2}:\frac{|h_{s,pi}^{H}w_{i}{|}^{2}}{|h_{s,pi}^{H}w_{j}{|}^{2}+{\left|h_{s,pi}^{H}w_{3}\right|}^{2}+\sigma_{p}^{2}}\geq z_{pi,2},\;i=1,2\\ & C_{3}:∥w_{1}{∥}^{2}+∥w_{2}{∥}^{2}+{∥w_{3}∥}^{2}\leq P_{s}, \end{array}$$

where $P_{s}=P_{\mathrm{th}}+Q\left(\beta^{★}\right)$ is the available transmit power at ST after energy harvesting.

### 3.2. Optimal Precoding Vectors for Spectrum Cooperation

To solve $Q_{1}$, we establish the following contrapositive of the problem:

(16) $$\begin{array}{cc} F_{1}:\;f\left(z_{s}\right)= & \min_{w_{i}}∥w_{1}{∥}^{2}+∥w_{2}{∥}^{2}+{∥w_{3}∥}^{2}\\ s.t. & \frac{|h_{s,pi}^{H}w_{i}{|}^{2}}{|h_{s,pi}^{H}w_{j}{|}^{2}+{\left|h_{s,pi}^{H}w_{3}\right|}^{2}+\sigma_{p}^{2}}\geq z_{pi,2},\\ & \;\;\;\;\;\;\;i,j=1,2,j\neq i \end{array}$$

(17) $$\begin{array}{cc} & \frac{|h_{s,s}^{H}w_{3}{|}^{2}}{\sum_{i=1,2}{\left|h_{s,s}^{H}w_{i}\right|}^{2}+\sigma_{s}^{2}}\geq z_{s}. \end{array}$$

The formulated contrapositive problem $F_{1}$ aims at minimizing the transmit power consumption at ST subject to both minimum rate requirements for PUs, i.e., $z_{pi,2}$, and minimum rate requirement for SU, i.e., $z_{s}$. Obviously, a bigger $z_{s}$ results in a bigger consumed power $f(z_{s})$. Thus, $f(z_{s})$ is also a monotonically increasing function about $z_{s}$, and the maximum achievable SU’s transmit data SINR $z_{s}$ for problem $Q_{1}$ can be solved by means of the bisection search method. Specifically, first solve the contrapositive problem $F_{1}$ for fixed $z_{s}$, and then compare the resultant minimum consumed power $f(z_{s})$ with the predetermined threshold value $P_{s}$ to update the searching region of $z_{s}$ until converges.

Work [32] s recommended for a better understanding of the bisection search methods. In what follows, the algorithm design for the contrapositive problem $F_{1}$ is illustrated. Note that, although problem $F_{1}$ is non-convex, it can be transformed into a second order cone programming problem, and hence its duality gap is zero. Therefore, we refer to the dual method to solve problem $F_{1}$.

First, introduce Lagrange multipliers $\lambda_{i},i=1,2,3$, and define the following Lagrangian function.

(18) $$\begin{array}{ccc} L & = & \sum_{i=1,2,3}{∥w_{i}∥}^{2}-\sum_{i=1,2}\lambda_{i}\left[|h_{s,pi}^{H}w_{i}{|}^{2}-z_{pi,2}\left(\right|h_{s,pi}^{H}w_{j}{|}^{2}+|h_{s,pi}^{H}w_{3}{|}^{2}+\sigma_{p}^{2})\right]\\ & & -\lambda_{3}\left[|h_{s,s}^{H}w_{3}{|}^{2}-z_{s}(\sum_{i=1,2}|h_{s,s}^{H}w_{i}{|}^{2}+\sigma_{s}^{2})\right],\;j\neq i. \end{array}$$

Its dual problem can be presented as follows:

(19) $$\begin{array}{cc} F_{1}^{\mathrm{Dual}} & \max_{\lambda_{i}\geq 0,1\leq i\leq 3}\sigma_{p}^{2}\sum_{i=1,2}\lambda_{i}z_{pi,2}+\lambda_{3}z_{s}\sigma_{s}^{2}\\ s.t. & I+\lambda_{j}z_{pj,2}h_{s,pj}h_{s,pj}^{H}+\lambda_{3}z_{s}h_{s,s}h_{s,s}^{H}⪰\lambda_{i}h_{s,pi}h_{s,pi}^{H},\\ & \;\;\;\;\;\;\;\;\;\;j\neq i,i=1,2\\ & I+\sum_{i=1,2}\lambda_{i}z_{pi,2}h_{s,pi}h_{s,pi}^{H}⪰\lambda_{3}h_{s,s}h_{s,s}^{H}. \end{array}$$

For the second constraint of the problem, it is easy to achieve that

(20) $$\begin{array}{c} \lambda_{3}\leq \frac{1}{h_{s,s}^{H}{\left[I+\sum_{i=1,2}\lambda_{i}z_{pi,2}h_{s,pi}h_{s,pi}^{H}\right]}^{-1}h_{s,s}}. \end{array}$$

Similarly, from the first constraint, it can be derived that

(21) $$\begin{array}{c} \lambda_{i}\leq \frac{1}{h_{s,pi}^{H}{\left[I+\lambda_{j}z_{pj,2}h_{s,pj}h_{s,pj}^{H}+\lambda_{3}z_{s}h_{s,s}h_{s,s}^{H}\right]}^{-1}h_{s,s}},\;j\neq i,i=1,2 \end{array}$$

According to Equations (20) and (21), the optimal $\lambda_{i}^{★},i=1,2,3$ can be obtained through a fixed point iteration algorithm [33].

Since the duality gap between problem $F_{1}$ and $F_{1}^{\mathrm{Dual}}$ is zero, hence after obtaining optimal $\lambda_{i}^{★},i=1,2,3$, the minimum transmit power consumption of problem $F_{1}^{\mathrm{Dual}}$ can be calculated as

(22) $$\begin{array}{c} f\left(z_{s}\right)=\sigma_{p}^{2}\sum_{i=1,2}\lambda_{i}^{★}z_{pi,2}+\lambda_{3}^{★}z_{s}\sigma_{s}^{2}. \end{array}$$

Meanwhile, the optimal encoding vectors for the primal problem can be related as

(23) $$\begin{array}{c} w_{i}^{★}=\sqrt{\chi_{i}}{\tilde{w}}_{i},\;i=1,2,3, \end{array}$$

where ${\tilde{w}}_{i}$ are normalized precoding vectors and $\chi_{i}$ is the corresponding power allocation. The normalized precoding vectors ${\tilde{w}}_{i}$ can be computed according to the uplink-downlink duality [31], expressed as

(24) $$\begin{array}{ccc} {\tilde{w}}_{i} & = & \frac{{\hat{w}}_{i}}{∥{\hat{w}}_{i}∥},{\hat{w}}_{i}={\left(I+\lambda_{j}z_{pj,2}h_{s,pj}h_{s,pj}^{H}+\lambda_{3}z_{s}h_{s,s}h_{s,s}^{H}\right)}^{-1}h_{s,pi},j\neq i,i=1,2,\\ {\tilde{w}}_{3} & = & \frac{{\hat{w}}_{3}}{∥{\hat{w}}_{3}∥},{\hat{w}}_{3}={\left(I+\sum_{i=1,2}\lambda_{i}z_{pi,2}h_{s,pi}h_{s,pi}^{H}\right)}^{-1}h_{s,s} \end{array}$$

and the power allocation $\chi_{i}$ can be computed according to the SINR constraints in problem $F_{1}$. That is, the optimal $\chi_{i}$ should satisfy the receiving SINR constraints in problem $F_{1}$, and hence can be computed as follows:

(25) $$\begin{array}{c} {\left[\chi_{1},\chi_{2},\chi_{3}\right]}^{T}=X^{-1}Y, \end{array}$$

where $\begin{array}{ccc} X & = & \left[\begin{array}{ccc} |h_{s,p1}^{H}{\tilde{w}}_{1}{|}^{2} & -z_{p1,2}{\left|h_{s,p1}^{H}{\tilde{w}}_{2}\right|}^{2} & -z_{p1,2}{\left|h_{s,p1}^{H}{\tilde{w}}_{3}\right|}^{2}\\ -z_{p2,2}{\left|h_{s,p2}^{H}{\tilde{w}}_{1}\right|}^{2} & |h_{s,p2}^{H}{\tilde{w}}_{2}{|}^{2} & -z_{p2,2}{\left|h_{s,p2}^{H}{\tilde{w}}_{3}\right|}^{2}\\ -z_{s}{\left|h_{s,s}^{H}{\tilde{w}}_{1}\right|}^{2} & -z_{s}{\left|h_{s,s}^{H}{\tilde{w}}_{2}\right|}^{2} & |h_{s,s}^{H}{\tilde{w}}_{3}{|}^{2} \end{array}\right]\;\mathrm{and}\;Y=\left[\begin{array}{c} z_{p1,2}\sigma_{p}^{2}\\ z_{p2,2}\sigma_{p}^{2}\\ z_{s}\sigma_{s}^{2} \end{array}\right]. \end{array}$

In conclusion, the solving procedure for problem $Q_{0}$ is summarized as follows. Firstly, compute the optimal PS factor $\beta^{★}$, i.e., the minimum $\beta^{★}$ satisfying two PUs’ minimum transmission rate requirements, and then acquire the simplified optimization problem $Q_{1}$. Secondly, establish the contrapositive problem $F_{1}$ for fixed SINR threshold of SU $z_{s}$, and obtain the optimal solution and the minimum transmit power consumption at ST, i.e., $w_{i}^{★},i=1,2,3$ and $f(z_{s})$ through the dual decomposition method. Finally, update searching range of $z_{s}$ by using the bisection method until the minimal power consumption $f(z_{s})$ equals to the maximum available transmit power at ST after energy harvesting, i.e., $P_{s}$. The detailed process is listed in Algorithm 1.

**Algorithm 1** Algorithm for solving problem $Q_{0}$.**Input:**  1: Basic parameters $N,\;\Delta f,\;\eta ,\;P_{i},i=1,2,\;P_{\mathrm{th}}$, channel parameters $h_{pi,pj},\;h_{pi,s},\;h_{s,pi},\;h_{s,s},\;i,j=1,2$,      noise parameters $\sigma_{p}^{2},\;\sigma_{s}^{2},\;\sigma_{a}^{2},\;\sigma_{b}^{2}$, and PUs’ minimum transmit rate requirements $R_{pi,\mathrm{th}},i=1,2$.**Implement:**  2: According to Equations (5) and (14), employ the bisection search method to compute $\beta_{\min,i},i=1,2$ and $\beta^{★}$.      Then establish the simplified problem $Q_{1}$.  3: Initialize the upper and lower bound of $z_{s}$ as $z_{s}^{\mathrm{up}}$, $z_{s}^{\mathrm{low}}$, and set the tolerated error as $\epsilon_{1}$.**Iteration:**  4: **while**
$z_{s}^{\mathrm{up}}-z_{s}^{\mathrm{low}}>\epsilon_{1}$
**do**  5:    Let $z_{s}=\left(z_{s}^{\mathrm{up}}+z_{s}^{\mathrm{low}}\right)/2$, and establish contrapositive problem $F_{1}$.  6:    Solve the contrapositive problem $F_{1}$ using dual decomposition method:        1> Build the dual problem $F_{1}^{\mathrm{Dual}}$ and derive the optimal dual variables $\lambda_{i}^{★}$ according to Algorithm 2;        2> Compute $w_{i},i=1,2,3$ and minimal power consumption $f(z_{s})$ based on Equations (22) and (23).  7:    If $f\left(z_{s}\right)>P_{s}$, set $z_{s}^{\mathrm{up}}=z_{s}$; Else, set $z_{s}^{\mathrm{low}}=z_{s}$.  8:  **end while****Output:**  9: Output SU’s maximal achievable transmit rate $R_{s}=\frac{1}{2}\Delta f\log_{2}\left(1+z_{s}\right)$.

**Algorithm 2** Fixed-point iteration algorithm of $\lambda_{i},i=1,2,3$ for problem $F_{1}^{\mathrm{Dual}}$.    **Initialization:**      1: Initialize t=0 and the initial dual variables $\lambda_{i}\left(0\right),i=1,2,3$.      2: Set the tolerated error as $\epsilon_{2}$ and any initial value $\Delta >\epsilon_{2}$.           **Iteration:**      3: **while**
$\Delta >\epsilon_{2}$
**do**      4:  Set t=t+1.      5:  Update $\lambda_{i}\left(t\right),i=1,2,3$ according to Equations (20) and (21).      6:  Compute $\Delta =\sum_{i=1,2,3}\left|\lambda_{i}\left(t\right)-\lambda_{i}\left(t-1\right)\right|$.      7:  **end while**

### 3.3. Complexity Analysis

According to the analysis above, the presented algorithm consists of two parts. The first one is to determine the optimal power splitting factor β. The update of β requires $\log_{2}\left(\frac{1}{\epsilon_{\beta }}\right)$ iterations, where $\epsilon_{\beta }$ is the tolerated error for β optimization. In each iteration, $R_{pi,1},i=1,2$ should be computed. The computation includes one matrix inversion operation $A^{-1}$ and one matrix multiplication operation $h_{pi,s}^{H}A^{-1}h_{pi,s}$, and the complexity is $O(N^{3})$ and $O(N^{2}),$ respectively. Hence, the overall complexity is $O\left\{N^{3}\log_{2}\left(\frac{1}{\epsilon_{\beta }}\right)\right\}$ for the optimal β determination. The second one is to optimize the precoding vectors. The outer bi-section search requires $\log_{2}\left(\frac{z_{s}^{\mathrm{up}}-z_{s}^{\mathrm{low}}}{\epsilon_{1}}\right)$ iterations, and the inner problem solving includes a fixed-point iteration of $\lambda_{i},i=1,2,3$ and for each iteration, some matrix operations are required. Taking $\lambda_{3}$ for example, the computation includes two matrix multiplication operations $h_{s,p,i}h_{s,p,i}^{H},i=1,2$, one matrix inversion operation $\left[I+\sum_{i=1,2}\right]\lambda_{i}z_{pi,s}h_{s,p,i}h_{s,p,i}^{H}$ and one matrix multiplication operation $h_{s,s}^{H}Bh_{s,s}$, and hence the required complexity is also $O(N^{3})$. As a result, the complexity of the second part is $O\left\{N^{3}N_{\lambda }\log_{2}\left(\frac{z_{s}^{\mathrm{up}}-z_{s}^{\mathrm{low}}}{\epsilon_{1}}\right)\right\}$, where $N_{\lambda }$ is the required number of fixed-point iterations for convergence. To sum up, the complexity of the whole algorithm could be expressed as $O\left\{N^{3}\left[\log_{2}\left(\frac{1}{\epsilon_{\beta }}\right)+N_{\lambda }\log_{2}\left(\frac{z_{s}^{\mathrm{up}}-z_{s}^{\mathrm{low}}}{\epsilon_{1}}\right)\right]\right\}$.

## 4. NC-Assisted Energy and Spectrum Dual-Cooperation for Symmetrical Primary Transmission

In this section, an NC-Assisted two-to-one cognitive cooperation is investigated with energy and spectrum dual-cooperation, in which two primary transmissions are symmetry.

### 4.1. System Model for Symmetrical Primary Transmission

In practice, the antenna number, or more accurately the dimensionality of effective channels, may be insufficient due to the small node size of ST, which often happens in wireless sensor networks. Under this circumstance, the spatial multiplexing freedom at ST may not be not enough to support simultaneous effective data transmission for two PUs and one SU. To cope with this issue, network coding function is introduced to reduce the resource consumption for signal forwarding at ST, alleviating the burden of spatial multiplexing. Meanwhile, according to the research on NC in traditional cooperation networks [24,25], it is the necessary condition that NC is preferable to that the transmission is symmetrical. As a result, we present the NC-assisted ESDC scheme for two-to-one cognitive cooperation to deal with the scenario of insufficient antenna number at ST and symmetrical primary transmission.

**Figure 2:**
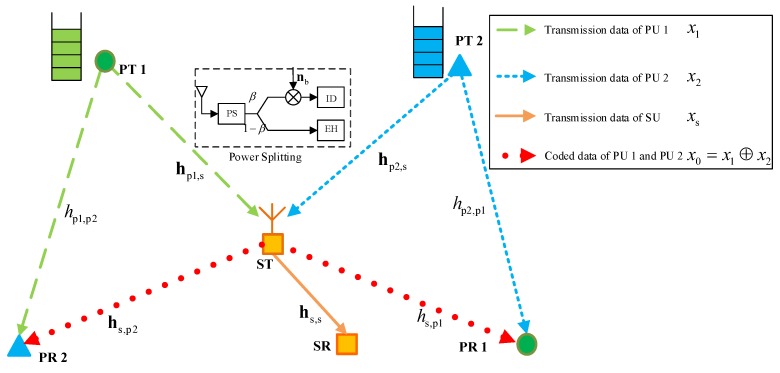
System model of two-to-one network coding (NC)-assisted energy and spectrum cooperation in cognitive cooperation networks.

Herein, the meaning of symmetrical primary transmission is two fold, as shown in Figure 2. Firstly, the two primary transmit-receive pairs must confirm to a so-called “butterfly topology” [25]. That is, both PTs are far from their own receivers and near to the receiver of the other one. Secondly, the required transmission data rates of two PUs must be close from each other. Note that the first condition is required such that each PR can successfully receive the transmission data of the other PT in the first slot, and hence be capable to perform the XOR operation after received the coded data from ST in the second slot. The second condition is also necessary from the perspective of maximizing performance gain of NC, which is briefly explained as follows. For an NC-assisted ESDC model, the transmission data of two PUs are recoded into one data stream through zero-padding and bitwise XOR operation, and the recoded data stream is transmitted to two PRs in a broadcasting manner. Clearly, the broadcasting of one recoded data stream is preferable over transmitting two data streams only when the transmission date of two users are close to each other.

Figure 2 is an illustration of the system model for the studied NC-assisted ESDC scheme. The whole transmission is also completed in two time slots. In the first slot, PTs broadcasts their message to both ST and PRs. At the side of ST, the received signal is passed through a power splitter to support simultaneously information decoding and energy harvesting. The corresponding signal process is the same as that in Section 2, and the overall harvested energy at ST and achievable receiving rate for PUs are shown in Equations (3) and (5). The only difference is that in slot one, each PR does not decode its intended signal, but the transmitted signal of the other PT to assist the data recovery from the received recoded data in slot two. Thus, the receiving rate for PU *i*’s data at PR *j* (j≠i,i,j=1,2) is

(26) $$\begin{array}{c} R_{pi,pj}=\frac{1}{2}\Delta f\log_{2}\left(1+\gamma_{pi,pj}\right),\;\gamma_{pi,pj}=\frac{P_{i}{\left|h_{pi,pj}\right|}^{2}}{P_{j}{\left|h_{pj,pj}\right|}^{2}+\sigma_{p}^{2}}. \end{array}$$

In the second slot, for decoded data $x_{1}$ and $x_{2}$, ST pads the short sequence with zero bits and then performs bitwise XOR operation to obtain the final transmitted data $x_{0}$. Afterwards, the coded data $x_{0}$ and SU’s own data $x_{s}$ are transmitted jointly through spatial multiplexing, whose precoding vectors are $w_{1}\in C^{N\times 1}$ and $w_{2}\in C^{N\times 1},$ respectively. Therefore, the final transmitted signal at ST is $s=w_{1}x_{0}+w_{2}x_{s}$, and the actually total consumed power is

(27) $$\begin{array}{c} P_{c}=∥w_{1}{∥}^{2}+{∥w_{2}∥}^{2}-Q\left(\beta \right). \end{array}$$

At PRs, the received signals and corresponding receiving rate for the coded data $x_{0}$, respectively, are

(28) $$\begin{array}{ccc} y_{\mathrm{PR}i,2} & = & h_{s,pi}^{H}s+n_{pi,2}=h_{s,pi}^{H}\left(w_{1}x_{0}+w_{2}x_{s}\right)+n_{pi}, \end{array}$$

(29) $$\begin{array}{ccc} R_{\mathrm{PR}i,2} & = & \frac{1}{2}\Delta f\log_{2}\left(1+\gamma_{pi,2}\right),\gamma_{pi,2}=\frac{|h_{s,pi}^{H}w_{1}{|}^{2}}{|h_{s,pi}^{H}w_{2}{|}^{2}+\sigma_{p}^{2}}. \end{array}$$

Similarly, at SR, the received signal and the receiving rate of data $x_{s}$, respectively, are

(30) $$\begin{array}{ccc} y_{\mathrm{SR}} & = & h_{s,s}^{H}s+n_{s,2}=h_{s,s}^{H}\left(w_{1}x_{0}+w_{2}x_{s}\right)+n_{s}, \end{array}$$

(31) $$\begin{array}{ccc} R_{s} & = & \frac{1}{2}\Delta f\log_{2}\left(1+\gamma_{s}\right),\;\gamma_{s}=\frac{|h_{s,s}^{H}w_{2}{|}^{2}}{|h_{s,s}^{H}w_{1}{|}^{2}+\sigma_{s}^{2}}. \end{array}$$

### 4.2. Problem Formulation for Symmetrical Primary Transmission

According to the above analysis, the optimization model for NC-assisted ESDC can be formulated in a similar manner to that for ESDC, as shown below:

(32) $$\begin{array}{cc} Q_{0}^{\mathrm{NC}}: & \max_{w_{1},w_{2},\beta }\gamma_{s}\\ s.t. & C_{1}:\gamma_{pi,1}\geq z_{pi,1},\;i=1,2,\\ & C_{2}:\gamma_{pi,2}\geq z_{p},\;i=1,2,\\ & C_{3}:P_{c}\leq P_{\mathrm{th}},\\ & C_{4}:\gamma_{pi,pj}\geq z_{pi,1},\;j\neq i,i,j=1,2, \end{array}$$

where $z_{p}=\max\left\{z_{p1,1},z_{p2,1}\right\}$ is required transmission SINR of the final recoded data for two PUs. Compared with problem $Q_{0}$, the receiving rate requirement at PR in the second slot, i.e., constraint $C_{2}$, is changed, which restricts the minimum receiving rate of PRs no less than the size of coded data $x_{0}$ at ST. Meanwhile, an extra constraint $C_{4}$ is added, which requires PR *j* to successfully decode PU *i*’s transmission data $x_{i}$ during the first slot, to recover its own data $x_{j}$ using XOR operation after receiving coded data $x_{0}$ in slot two.

### 4.3. Algorithm Design for NC-Assisted ESDC

It is easily found that the constraint $C_{4}$ in problem $Q_{0}^{\mathrm{NC}}$ is irrelevant to the optimization of $w_{1}$, $w_{2}$ and β, and can be verified in advance. Obviously, the NC-assisted ESDC is meaningful only when constraint $C_{4}$ is satisfied. Hence, in the following algorithm design, constraint $C_{4}$ is deemed as satisfied and hence omitted.

Moreover, similar to the analysis in Section 3, there is a trade-off of the value of PS factor to balance the signal decoding in the first slot and data delivery in the second slot. Since the ultimate harvested energy and achievable receiving rate for PUs’ data at ST remains the same as their counterparts in ESDC model. The optimal PS factor $\beta^{★}$ can also be determined according to Equation (14). In a consequence, problem $Q_{0}^{\mathrm{NC}}$ is simplified as

(33) $$\begin{array}{cc} Q_{1}^{\mathrm{NC}}: & \max_{w_{1},w_{2}}\frac{|h_{s,s}^{H}w_{2}{|}^{2}}{|h_{s,s}^{H}w_{1}{|}^{2}+\sigma_{s}^{2}}\\ s.t. & \frac{|h_{s,pi}^{H}w_{1}{|}^{2}}{|h_{s,pi}^{H}w_{2}{|}^{2}+\sigma_{p}^{2}}\geq z_{p},\;i=1,2\\ & ∥w_{1}{∥}^{2}+{∥w_{2}∥}^{2}\leq P_{s}. \end{array}$$

To solve the problem, we build the following contrapositive power minimization problem, in which we fix the receiving SINR for SU’s data at SR as $z_{s}$ and then optimize the precoding vectors $w_{1},w_{2}$ to minimize the transmit power consumption at ST:

(34) $$\begin{array}{cc} F_{1}^{\mathrm{NC}}:\;f^{\mathrm{NC}}\left(z_{s}\right)= & \min_{w_{1},w_{2}}∥w_{1}{∥}^{2}+{∥w_{2}∥}^{2}\\ s.t. & \frac{|h_{s,pi}^{H}w_{1}{|}^{2}}{|h_{s,pi}^{H}w_{2}{|}^{2}+\sigma_{p}^{2}}\geq z_{p},\;i=1,2\\ & \frac{|h_{s,s}^{H}w_{2}{|}^{2}}{|h_{s,s}^{H}w_{1}{|}^{2}+\sigma_{s}^{2}}\geq z_{s}. \end{array}$$

Obviously, along with the increase of $z_{s}$, the objective function ST $f^{\mathrm{NC}}\left(z_{s}\right)$ increases monotonically. Thus, $f^{\mathrm{NC}}\left(z_{s}\right)$ is a monotonically increasing function about $z_{s}$, and the maximum achievable SU’s transmit data SINR $z_{s}$ for problem $Q_{1}^{\mathrm{NC}}$ can be solved by means of the bisection search method, similar to the process for problem $Q_{1}$ and $F_{1}$.

In the following, how to solve the contrapositive problem $F_{1}^{\mathrm{NC}}$ is described in detail. Since problem $F_{1}^{\mathrm{NC}}$ is not and even cannot be transformed into a convex problem, the dual method is not competent. In the following, we refer to the semi-definitive relation (SDR) method. Firstly, define $W_{i}=w_{i}w_{i}^{H},i=1,2$, problem $F_{1}^{\mathrm{NC}}$ can be converted as follows:

(35) $$\begin{array}{cc} F_{2}^{\mathrm{NC}}: & \min_{W_{i},i=1,2}\mathrm{Tr}\left(W_{1}\right)+\mathrm{Tr}\left(W_{2}\right)\\ s.t. & \mathrm{Tr}\left(h_{s,pi}h_{s,pi}^{H}W_{1}\right)\geq z_{p}\left[\mathrm{Tr}\left(h_{s,pi}h_{s,pi}^{H}W_{2}\right)+\sigma_{p}^{2}\right],\;i=1,2\\ & \mathrm{Tr}\left(h_{s,s}h_{s,s}^{H}W_{2}\right)\geq z_{s}\left[\mathrm{Tr}\left(h_{s,s}h_{s,s}^{H}W_{1}\right)+\sigma_{s}^{2}\right]\\ & W_{i}⪰0,\;\mathrm{Rank}\left(W_{i}\right)=1,\;i=1,2. \end{array}$$

Problem $F_{2}^{\mathrm{NC}}$ is non-convex due to the rank-one constraint, i.e., $\mathrm{Rank}(W_{i})=1,i=1,2$. To circumvent this, we refer to the Semi-Definite Positive Relation (SDR) [34], and drop the rank-one constraint to simplify the problem as follows:

(36) $$\begin{array}{cc} F_{2,\mathrm{Relax}}^{\mathrm{NC}}: & \min_{W_{i},i=1,2}\mathrm{Tr}\left(W_{1}\right)+\mathrm{Tr}\left(W_{2}\right)\\ s.t. & \mathrm{Tr}\left(h_{s,pi}h_{s,pi}^{H}W_{1}\right)\geq z_{p}\left[\mathrm{Tr}\left(h_{s,pi}h_{s,pi}^{H}W_{2}\right)+\sigma_{p}^{2}\right],i=1,2\\ & \mathrm{Tr}\left(h_{s,s}h_{s,s}^{H}W_{2}\right)\geq z_{s}\left[\mathrm{Tr}\left(h_{s,s}h_{s,s}^{H}W_{1}\right)+\sigma_{s}^{2}\right]\\ & W_{i}⪰0,\;i=1,2. \end{array}$$

The problem is a semi-definite programming (SDP) problem and can be solved by convex optimization algorithm, such as the inner-point method in polynomial time [35]. The details are ignored here due to space limitation, and, in the presented simulations in Section 5, the relaxed problem is solved by toolkit build-in cvx of MATLAB R2014a. In the following, the resultant optimal solution for problem $F_{2,\mathrm{Relax}}^{\mathrm{NC}}$ is denoted as $W_{i}^{★},i=1,2$.

However, since the rank-one constraint is not included in problem $F_{2,\mathrm{Relax}}^{\mathrm{NC}}$, the obtained solution $W_{i}^{★},i=1,2$ may not satisfy the rank-one constraint. However, through an in-depth analysis, it is found that the solution is feasible in a sense that we can find at least one equivalent solution to $W_{i}^{★},i=1,2$ which satisfies the rank-one solution and results in the same power consumption at ST.

**Theorem** **1.** For problem $F_{2,\mathrm{Relax}}^{\mathrm{NC}}$, the following properties hold: (1) Its optimal solution $W_{i}^{★},i=1,2$ satisfies $Rank[W_{1}^{★}]\leq 2$, $Rank[W_{2}^{★}]=1$; (2) There must be at least one optimal solution satisfying $Rank[{\tilde{W}}_{1}^{★}]=1$ and $Rank[W_{2}^{★}]=1$.

**Proof** **of** **Theorem** **1.** See Appendix A. ☐

As a result, the solution $W_{i}^{★},i=1,2$ must satisfy $\mathrm{Rank}[W_{2}^{★}]=1$. In the case that $\mathrm{Rank}[W_{1}^{★}]=2$, the equivalent solution satisfying rank-one constraint can be derived by purification technology. Detailed process is omitted due to space limitation, and work [36] is recommended for better understanding. Finally, after obtaining the optimal $W_{i}^{★},i=1,2$ of rank-one property, the corresponding precoding vectors $w_{i}^{★},i=1,2$ can be easily derived from the singular value decomposition (SVD) of $W_{i}^{★},i=1,2$.

In conclusion, the solving process for the optimization problem $Q_{0}^{\mathrm{NC}}$ in an NC-assisted ESDC model can be summarized as follows. Firstly, compute the optimal PS factor $\beta^{★}$, i.e., the minimum $\beta^{★}$ satisfying two PUs’ minimum transmission rate requirements, and then acquire the simplified optimization problem $Q_{1}^{\mathrm{NC}}$. Secondly, establish the contrapositive problem $F_{1}^{\mathrm{NC}}$ for fixed SINR threshold of SU $z_{s}$, and obtain the optimal solution and the minimum transmit power consumption at ST, i.e., $w_{i},i=1,2$ and $f^{\mathrm{NC}}\left(z_{s}\right)$ through SDP and the purification process. Finally, update searching range of $z_{s}$ using the bisection method until the minimal power consumption $f^{\mathrm{NC}}\left(z_{s}\right)$ equals to the maximum available transmit power at ST after energy harvesting, i.e., $P_{s}$. The detailed process is listed in Algorithm 3.

**Algorithm 3** Algorithm for solving problem $Q_{0}^{\mathrm{NC}}$.**Input:**  1: Basic parameters $N,\;\Delta f,\;\eta ,\;P_{i},i=1,2,\;P_{\mathrm{th}}$, channel parameters $h_{pi,pj},\;h_{pi,s},\;h_{s,pi},\;h_{s,s},\;i,j=1,2$,      noise parameters $\sigma_{p}^{2},\;\sigma_{s}^{2},\;\sigma_{a}^{2},\;\sigma_{b}^{2}$, and PUs’ minimum transmit rate requirements $R_{pi,\mathrm{th}},i=1,2$.**Implement:**  2: According to Equations (5) and (14), employ the bisection search method to compute $\beta_{\min,i},i=1,2$ and $\beta^{★}$.      Then establish the simplified problem $Q_{1}^{\mathrm{NC}}$.  3: Initialize the upper and lower bound of $z_{s}$ as $z_{s}^{\mathrm{up}}$, $z_{s}^{\mathrm{low}}$, and set the tolerated error as $\epsilon_{3}$.**Iteration:**  4:  **while**
$z_{s}^{\mathrm{up}}-z_{s}^{\mathrm{low}}>\epsilon_{3}$
**do**  5:    Let $z_{s}=\left(z_{s}^{\mathrm{up}}+z_{s}^{\mathrm{low}}\right)/2$, and establish contrapositive problem $F_{1}^{\mathrm{NC}}$.  6:    Using SDP and purification technology if necessary to solve problem $F_{1}^{\mathrm{NC}}$ and obtain the optimal            solution $w_{i}^{★},i=1,2$ and the minimum required transmit power $f^{\mathrm{NC}}\left(z_{s}\right)$.  7:    If $f^{\mathrm{NC}}\left(z_{s}\right)>P_{s}$, set $z_{s}^{\mathrm{up}}=z_{s}$; Else, set $z_{s}^{\mathrm{low}}=z_{s}$.  8:  **end while****Output:**  9:  Output SU’s maximum achievable transmit rate $R_{s}=\frac{1}{2}\Delta f\log_{2}\left(1+z_{s}\right)$.

### 4.4. Complexity Analysis

Similar to the analysis in Section 3.3, the presented algorithm consists of two parts. The first one is to determine the optimal power splitting factor β and the complexity is $O\left\{N^{3}\log_{2}\left(\frac{1}{\epsilon_{\beta }}\right)\right\}$. The second one is to optimize the precoding vectors in which the outer bi-section search requires $\log_{2}\left(\frac{z_{s}^{\mathrm{up}}-z_{s}^{\mathrm{low}}}{\epsilon_{3}}\right)$ iterations and for each iteration, the complexity of solving problem $F_{1}^{\mathrm{NC}}$ using SDP is $O\left\{{\left(6+N^{2}\right)}^{3.5}\right\}$ [37]. As a result, the complexity of the second part is $O\left\{{\left(6+N^{2}\right)}^{3.5}\log_{2}\left(\frac{z_{s}^{\mathrm{up}}-z_{s}^{\mathrm{low}}}{\epsilon_{3}}\right)\right\}$. To sum up, the complexity of the whole algorithm could be expressed as $O\left\{N^{3}\log_{2}\left(\frac{1}{\epsilon_{\beta }}\right)+{\left(6+N^{2}\right)}^{3.5}\log_{2}\left(\frac{z_{s}^{\mathrm{up}}-z_{s}^{\mathrm{low}}}{\epsilon_{3}}\right)\right\}$.

## 5. Simulation Results

In the following simulations, the parameter settings are given in Table 1. In detail, the spectrum bandwidth is set as 2 MHz, and the number of antennas equipped at ST are N=4 or 2. The transmit powers at PT 1, PT 2 and ST are 1.5 W, 1.0 W and 0.2 W, respectively, and the energy conversion efficiency at ST is assumed as η=1. The power of receiving noise is set as $\sigma_{p1}^{2}=\sigma_{p2}^{2}=\sigma_{s}^{2}=\sigma_{n}^{2}$ dBW and $\sigma_{a}^{2}=\sigma_{b}^{2}=\sigma_{n}^{2}-3$ dBW by default.

Meanwhile, the transmission channel between any two transmit-receive antennas includes both large-scale fading and small-scale fading. The small fading is modeled as independent identically distributed Rayleigh variable with mean value of zero. The path loss for channel from PT 1 to PR 1/PR 2, from PT 2 to PR 2/PR 1, from PT 1/PT 2 to ST, and from ST to PR 1/PR 2/SR are set as −30 dB/−10 dB, −30 dB/−10 dB, −15 dB/−15 dB, and −15 dB/−15 dB/−15 dB, respectively. It is worth noting that the two transmit-receive nodes implicitly make up a “butterfly” structure with such a pathloss setting.

In the following text, six kinds of cognitive cooperation schemes are compared, in which both the successful transmission probability of PUs and the achievable transmission rate of SU are plotted with different parameter settings for the antenna number *N*, the target transmission rate of two PUs $R_{pi,\mathrm{th}},i=1,2$ and the receiving noise power $\sigma_{n}^{2}$. The involved simulation schemes are explained as follows:

- Two-to-one ESDC is the proposed two-to-one energy and spectrum dual-cooperation scheme. The corresponding problem formulation is $Q_{0}$ and the detailed algorithm design is explained in Algorithms 1 and 2.
- Two-to-one NC-ESDC is the proposed two-to-one NC-assisted energy and spectrum dual-cooperation scheme. The corresponding problem formulation is $Q_{0}^{\mathrm{NC}}$ and the detailed algorithm design is explained in Algorithm 3.
- Two-to-one SC is the two-to-one spectrum-only cooperation scheme, and can be deemed as a simplified version of “Two-to-one ESDC”, in which the energy cooperation is removed.
- Two-to-one NC-SC is the two-to-one NC-assisted spectrum-only cooperation scheme, and can be deemed as a simplified version of “Two-to-one NC-ESDC”, in which the energy cooperation is removed.
- One-plus-one ESDC (Single) is the one-plus-one energy and spectrum dual-cooperation scheme, in which SU random selects one PU to cooperate and the detailed algorithm design can be found in Section 4 of reference [15].
- One-plus-one ESDC (Both) is a modified version of “One-plus-one ESDC (Single)”, in which SU divides its transmission time and energy into two equal parts and cooperates with two PUs sequentially.

Note that all the obtained curves in the following figures are averaged over 2000 channel realizations.

**Figure 3:**
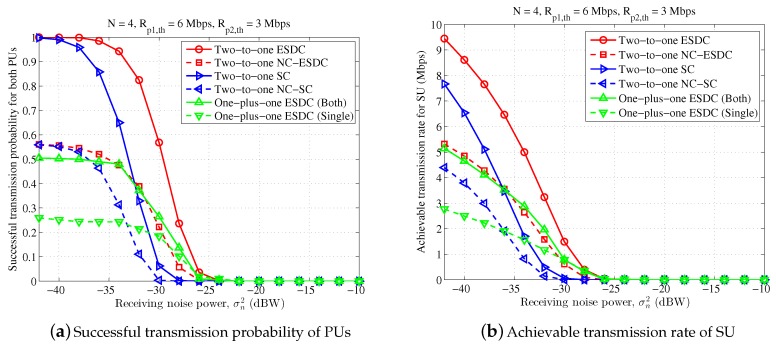
Performance comparison for different receiving noise power, in which n=4, $R_{p1,\mathrm{th}}=6\;\mathrm{Mbps}$, $R_{p2,\mathrm{th}}=3\;\mathrm{Mbps}$.

**Figure 4:**
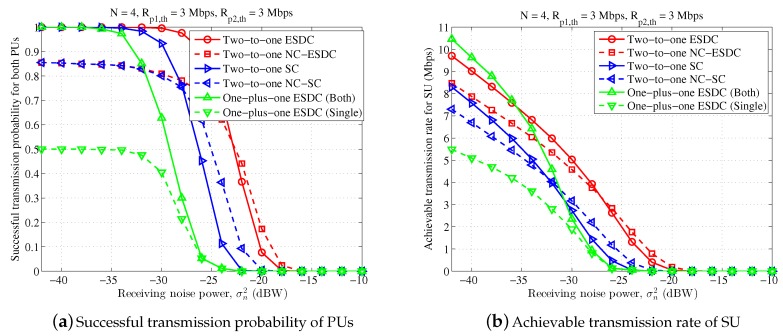
Performance comparison for different receiving noise power, in which n=4, $R_{p1,\mathrm{th}}=3\;\mathrm{Mbps}$, $R_{p2,\mathrm{th}}=3\;\mathrm{Mbps}$.

**Figure 5:**
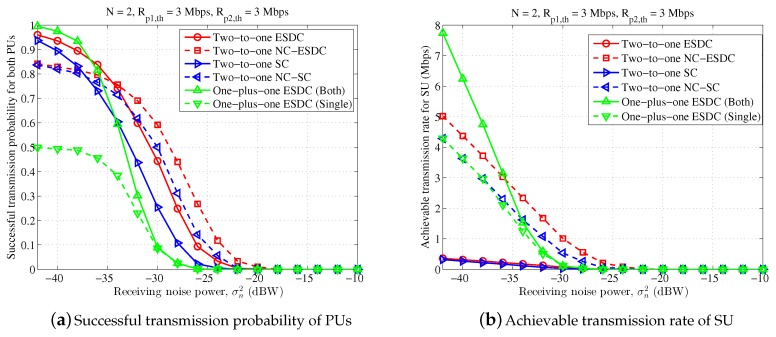
Performance comparison for different receiving noise power, in which n=2, $R_{p1,\mathrm{th}}=3\;\mathrm{Mbps}$, $R_{p2,\mathrm{th}}=3\;\mathrm{Mbps}$.

Figure 3, Figure 4 and Figure 5 depict average achieved performance for different schemes with different receiving noise power, ranging from −42 dBW to −10 dBW. In Figure 3, the antenna number at ST is set as 4 and the target transmission rates of two PUs are $R_{p1,\mathrm{th}}=6$ Mbps, $R_{p2,\mathrm{th}}=3$ Mbps. It can be found that, on one hand, the proposed energy and spectrum dual-cooperation scheme outperforms the simplified spectrum-only schemes in both cases of non-NC-assisted model and NC-assisted model. Taking the non-NC-assisted schemes for example, the average successful transmission probability of PUs and the achievable transmission rate of SU are improved by 34% and 193%, respectively, when $\sigma_{n}^{2}=-34$ dBW. One the other hand, the proposed two-to-one cooperation scheme is also superior than the existing one-plus-one cooperation scheme for the adopted parameter settings. For example, compared to the schemes “One-plus-one ESDC (Both)” and “One-plus-one ESDC (Single)”, the successful transmission probability of PUs obtained in the scheme “Two-to-one ESDC” increases by one-fold and three-fold, respectively, and the corresponding achievable transmission rate of SU is enhanced by 84% and 238% respectively, when $\sigma_{n}^{2}=-36$ dBW. Finally, it is also observed that the performance of the non-NC-assisted schemes are better than that of NC-assisted scheme for two-to-one cooperation in this figure. This is not beyond our expectation since the target transmission rates of two PUs are far from each other and the condition of symmetrical primary transmission is violated.

In Figure 4, we set $R_{p1,\mathrm{th}}=R_{p2,\mathrm{th}}=3$ Mbps to simulate a scene of symmetrical primary transmission. Similarly, the proposed two-to-one energy and spectrum dual-cooperation schemes outperform both the two-to-one spectrum-only cooperation schemes and the one-plus-one energy and spectrum dual-cooperation schemes for the majority range of $\sigma_{n}^{2}$. The exception happens in the region of small $\sigma_{n}^{2}$, or more accurately $\sigma_{n}^{2}\leq -36$ dBW, in which the scheme “One-plus-one ESDC (Both)” can support a higher transmission rate for SU than the proposed schemes “Two-to-one ESDC” and “Two-to-one NC-ESDC”, whilst remain the same high successful transmission probability for PUs. This observation suggests that for high transmission SINR, executing the three-party cooperation between one SU and two PUs simultaneously is not a good choice. By instead, it is more desired that SU cooperates with two PUs separately through time division. In the meantime, different from the results for the asymmetrical scene in Figure 3, the merits of NC-assisted schemes become larger and offset its demerits gradually when the transmission condition becomes worse and worse. Particularly, the performance of NC-assisted spectrum-only cooperation and energy and spectrum dual-cooperation schemes becomes larger than the corresponding non-NC-assisted schemes when $\sigma_{n}^{2}\geq -32$ dBW and $\sigma_{n}^{2}\geq -26$ dBW, respectively.

However, the performance gain of NC-assisted schemes over the non-NC-assisted schemes are not prominent in Figure 4. This is because the ST is equipped with four antennas and the spatial multiplexing capability is high enough to support effective data transmission for PU 1, PU 2 and SU, simultaneously. To verify the superiority of NC-assisted schemes, Figure 5 is demonstrated in which the antenna number at ST is set as n=2. In this circumstance, the spatial multiplexing capability at ST is greatly crippled, and ST can only support the data transmission for two PUs with little resources reserved for its own data transmission. As a result, the achievable transmission rate of SU is very small, below 0.4 Mbps even for high SINR. By contrast, the NC-assisted scheme successfully avoids this issue by recoding the two PUs’ data streams into one, and hence the corresponding transmission rate of SU becomes much higher.

**Figure 6:**
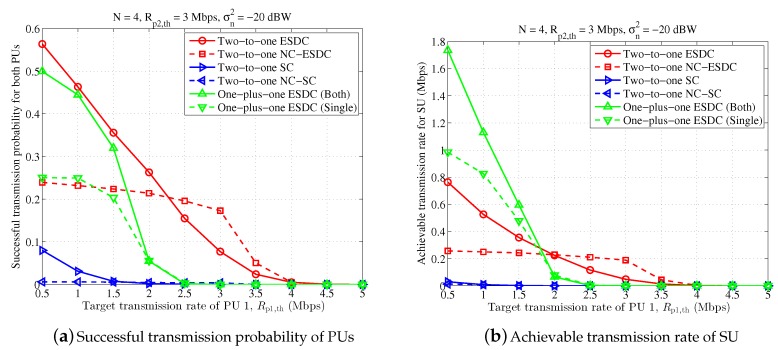
Performance comparison for different target transmission rates of primary user (PU) 1, in which n=4, $R_{p2,\mathrm{th}}=3\;\mathrm{Mbps}$, $\sigma_{n}^{2}=-20$ dBW.

**Figure 7:**
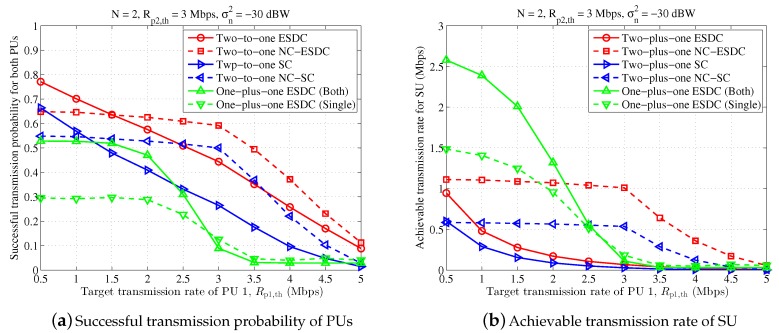
Performance comparison for different target transmission rates of PU 1, in which n=2, $R_{p2,\mathrm{th}}=3\;\mathrm{Mbps}$, $\sigma_{n}^{2}=-30$ dBW.

In Figure 6 and Figure 7, we further demonstrate the average successful probability of PUs and achievable transmission rate of SU when the target transmission rate of PU 1 changes. From these two figures, it is seen that the proposed energy and spectrum dual-cooperation schemes are always better than the simplified spectrum-only cooperation schemes. Moreover, the performance obtained in the proposed two-to-one cooperation schemes is better than that in the one-plus-one cooperation schemes for a larger target transmission rate $R_{p1,\mathrm{th}}$. With respect to the case of small $R_{p1,\mathrm{th}}$, the proposed two-to-one cooperation schemes are still more competent in successful transmission probability of PUs but loses the advantage in the achievable transmission rate of SU. Taken together with the observation in Figure 4 and Figure 5 that “One-plus-one ESDC (Both)” is desired for high transmission SINR, we can conclude that the one-plus-one cooperation schemes are more appealing when the transmission condition of PUs are good and the required transmission data size is small. Interestingly, under such circumstances, PUs are often capable of accomplishing their data transmission independently, making them less willing to cooperate with SU.

In addition, it is shown in Figure 6 that, compared with the non-NC-assisted schemes, the NC-assisted schemes perform better when the target transmission rate of PU 1 becomes close to that of PU 2. For instance, for $2.2\leq R_{p1,\mathrm{th}}\leq 4$ Mbps, both the achieved successful transmission probability of PUs and the achievable transmission rate of SU in the scheme “Two-to-one NC-ESDC” become higher than those obtained in the scheme “Two-to-one ESDC”. This performance gain is further enhanced in Figure 7 where two antennas are equipped at ST. These results prove the good applicability of NC-assisted schemes for symmetrical primary transmission once more.

**Figure 8:**
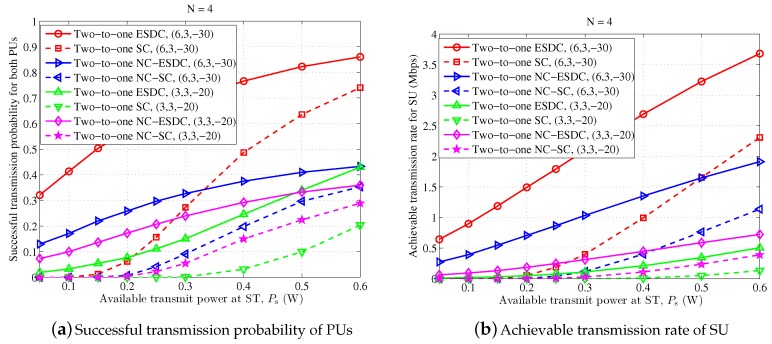
Performance comparison for different available battery power $P_{\mathrm{th}}$ at secondary transmitter (ST), in which n=4 and parameter setting for $R_{p1,\mathrm{th}}\;(\mathrm{Mbps})$, $R_{p2,\mathrm{th}}\;(\mathrm{Mbps})$ and $\sigma_{n}^{2}\;(\mathrm{dBW})$ are denoted as a triple (x,y,z) shown in the legends.

Finally, we return back the proposed two-to-one cooperation schemes, and illustrate the relationship between the available battery power $P_{s}$ and the performance gain brained by energy harvesting at ST, as shown in Figure 8. It is clearly shown that the energy and spectrum dual-cooperation schemes are superior to the spectrum-only cooperation schemes, and the performance gain is significantly high when the available battery power at ST is small. For instance, when $P_{\mathrm{th}}\leq 0.15$ W, the achieved successful transmission probability of PUs and average transmission rate of SU in the scheme “Two-to-one SC” nearly reaches zero. By contrast, the scheme “Two-to-one ESDC” still performs well, and the corresponding successful transmission probability of PUs and average transmission rate of SU remain higher than 33% and 0.65 Mbps. Meanwhile, along with the increase of the available battery power at ST, on one hand, the performance gain incurred by energy cooperation degrades accordingly for the successful transmission probability of PUs. Particularly, it is expected that when $P_{\mathrm{th}}$ becomes higher than some thresholds, the achieved successful transmission probability of PUs becomes saturated for both spectrum-only and energy and spectrum dual-cooperation schemes. On the other hand, the performance gain for achievable transmission rate of SU remains nontrivial, since the harvested energy is always meaningful to improve the data transmission of SU. In addition, by comparing the non-NC-assisted schemes, i.e., the solid red and green curves, and NC-assisted schemes, i.e., the solid blue and purple curves, it is observed that the non-NC-assisted schemes behave better in the case of asymmetrical primary transmission, while the NC-assisted schemes are more competent for the symmetrical primary transmission especially when the battery power at ST is relatively small.

## 6. Conclusions

In this paper, we have investigated the energy and spectrum dual-cooperation scheme for two-to-one cognitive cooperation. Therein, spatial multiplexing is integrated to enable simultaneous data forwarding for PUs and data transmission for SU, and network coding is employed as a complementary solution in the case of small antennas equipped at ST and symmetrical transmission for PUs, through which the resource consumption for PUs’ data forwarding is further reduced. For this kind of new cognitive cooperation model, the optimal algorithm design is proposed to acquire the optimal cooperation strategy. In detail, we first derive the minimum feasible PS factor to achieve the maximum performance gain from energy cooperation, and then design the optimal precoding vectors for both PUs’ and SU’s data transmission by means of the Lagrangian dual method (for non-NC-assisted model) and the SDR method (for NC-assisted model). Simulation results show that the proposed energy and spectrum dual-cooperation schemes significantly outperform the simplified spectrum-only cooperation schemes, and the proposed two-to-one cooperation model is more competent than the existing one-plus-one model for the majority of parameter settings. Moreover, it is also demonstrated that the proposed non-NC-assisted ESDC scheme behaves better in the case of asymmetrical primary transmission and the NC-assisted ESDC scheme is superior for the symmetrical primary transmission, especially when the node size of ST is small with a small number of equipped antennas. That is, these two schemes are complementary to each other, and hence an adaptive mode-switching between these two schemes may be a better choice in practice. However, only a perfect channel model is considered in this paper, and, in our future work, the imperfect channel model based on channel estimation and feedback will be considered to design a more realistic paradigm for energy and spectrum cooperation between PUs and SU.

## Figures and Tables

**Table 1:** Simulation parameters.

Parameter Name	Value
Spectrum bandwidth	Δf=2 MHz
Number of antennas equipped at ST	n= 4 or 2
Transmit power at PT 1/PT 2	$P_{1}=1.5$ W, $P_{2}=1$ W
Available transmit power at ST 1	$P_{\mathrm{th}}=0.2$ W
Energy conversion efficiency at ST	η=1
Power of receiving noise at PT 1/PT 2/ST	$\sigma_{p1}^{2}=\sigma_{p2}^{2}=\sigma_{s}^{2}=\sigma_{n}^{2}$ dBW
Power of receiving noise at ST from antennas	$\sigma_{a}^{2}=\sigma_{n}^{2}$ − 3 dBW
Noise power from RF/Baseband conversion and processing	$\sigma_{b}^{2}=\sigma_{n}^{2}$ − 3 dBW
Path loss for channel from PT 1 to PR 1/PR 2	$∥h_{p1,p1}{∥}^{2}=-30$ dB, $∥h_{p1,p2}{∥}^{2}=-10$ dB
Path loss for channel from PT 2 to PR 2/PR 1	$∥h_{p2,p2}{∥}^{2}=-30$ dB, $∥h_{p2,p1}{∥}^{2}=-10$ dB
Path loss for channel from PT 1/PT 2 to ST	$∥h_{p1,s}{∥}^{2}={∥h_{p2,s}∥}^{2}=-15$ dB
Path loss for channel from ST to PR 1/PR 2/SR	$∥h_{s,p1}{∥}^{2}=∥h_{s,p2}{∥}^{2}={∥h_{s,s}∥}^{2}=-15$ dB

## Appendix A. Proof of Theorem 1

For problem $F_{2,\mathrm{Relax}}^{\mathrm{NC}}$, define Lagrange multipliers $\lambda_{i},i=1,2,3$, and the Lagrange function is

(A1) $$\begin{array}{ccc} L & = & \sum_{i=1,2}\lambda_{i}z_{p}\sigma_{p}^{2}+\lambda_{3}z_{s}\sigma_{s}^{2}+\mathrm{Tr}\left[T_{1}W_{1}\right]+\mathrm{Tr}\left[T_{2}W_{2}\right], \end{array}$$

(A2) $$\begin{array}{ccc} T_{1} & = & I-\sum_{i=1,2}\lambda_{i}h_{s,pi}h_{s,pi}^{H}+\lambda_{3}z_{s}h_{s,s}h_{s,s}^{H}, \end{array}$$

(A3) $$\begin{array}{ccc} T_{2} & = & I+\sum_{i=1,2}\lambda_{i}z_{p}h_{s,pi}h_{s,pi}^{H}-\lambda_{3}h_{s,s}h_{s,s}^{H}. \end{array}$$

Let $W_{i}^{★},i=1,2$ and $\lambda_{i}^{★},i=1,2,3$ denote the optimal primary solution and dual solution, respectively. Then, according to KKT condition, one can obtain that $T_{i}^{★}W_{i}^{★}=0$. That is,

(A4) $$\begin{array}{c} \left(I+\lambda_{3}^{★}z_{s}h_{s,s}h_{s,s}^{H}\right)W_{1}^{★}=\sum_{i=1,2}\lambda_{i}^{★}h_{s,pi}h_{s,pi}^{H}W_{1}^{★}, \end{array}$$

(A5) $$\begin{array}{c} \left(I+\sum_{i=1,2}\lambda_{i}z_{p}h_{s,pi}h_{s,pi}^{H}\right)W_{2}^{★}=\lambda_{3}h_{s,s}h_{s,s}^{H}W_{2}^{★}. \end{array}$$

Because $\mathrm{Rank}[I+\lambda_{3}^{★}z_{s}h_{s,s}h_{s,s}^{H}]=N$ and $\mathrm{Rank}[I+\sum_{i=1,2}\lambda_{i}z_{p}h_{s,pi}h_{s,pi}^{H}]=N$, we have

(A6) $$\begin{array}{ccc} \mathrm{Rank}[W_{1}^{★}] & = & \mathrm{Rank}[\left(I+\lambda_{3}^{★}z_{s}h_{s,s}h_{s,s}^{H}\right)W_{1}^{★}]\\ & = & \mathrm{Rank}[\sum_{i=1,2}\lambda_{i}^{★}h_{s,pi}h_{s,pi}^{H}W_{1}^{★}]\\ & \leq & \mathrm{Rank}[\sum_{i=1,2}\lambda_{i}^{★}h_{s,pi}h_{s,pi}^{H}],\\ & \leq & 2 \end{array}$$

(A7) $$\begin{array}{ccc} \mathrm{Rank}[W_{2}^{★}] & = & \mathrm{Rank}[\left(I+\sum_{i=1,2}\lambda_{i}z_{p}h_{s,pi}h_{s,pi}^{H}\right)W_{2}^{★}]\\ & = & \mathrm{Rank}[\lambda_{3}h_{s,s}h_{s,s}^{H}W_{2}^{★}]\\ & \leq & \mathrm{Rank}[\lambda_{3}h_{s,s}h_{s,s}^{H}]\\ & \leq & 1. \end{array}$$

Obviously, $\mathrm{Rank}[W_{2}^{★}]=0$ cannot satisfy the minimum rate constraint of SU. Therefore, $\mathrm{Rank}[W_{1}^{★}]\leq 2$ and $\mathrm{Rank}[W_{2}^{★}]=1$.

Moreover, according to the conclusion of *Lemma 3.1* in [36], it is shown that there is at least one optimal solution satisfy

(A8) $$\begin{array}{c} \mathrm{Rank}{\left[W_{1}^{★}\right]}^{2}+\mathrm{Rank}{\left[W_{2}^{★}\right]}^{2}\leq 3. \end{array}$$

That is to say, there must be one optimal solution that satisfies $\mathrm{Rank}[W_{1}^{★}]\leq 1$. Similarly, $\mathrm{Rank}[W_{1}^{★}]=0$ makes no sense, and, thus, there is at least one optimal solution $W_{2}^{★}$ that satisfies $\mathrm{Rank}[W_{2}^{★}]=1$.

Until now, *Theorem 1* is proved.
